# The role of RIM in neurotransmitter release: promotion of synaptic vesicle docking, priming, and fusion

**DOI:** 10.3389/fnins.2023.1123561

**Published:** 2023-04-26

**Authors:** Shanshan Wu, Jiali Fan, Fajuan Tang, Lin Chen, Xiaoyan Zhang, Dongqiong Xiao, Xihong Li

**Affiliations:** ^1^Emergency Department, West China Second University Hospital, Sichuan University, Chengdu, China; ^2^Key Laboratory of Birth Defects and Related Diseases of Women and Children (Sichuan University), Ministry of Education, Chengdu, China

**Keywords:** cytomatrix in the active zone (CAZ), regulating synaptic membrane exocytosis protein (RIM), soluble N-ethylmaleimide-sensitive factor attachment protein receptor (SNARE), synaptic vesicles, neurotransmitter release

## Abstract

There are many special sites at the end of a synapse called active zones (AZs). Synaptic vesicles (SVs) fuse with presynaptic membranes at these sites, and this fusion is an important step in neurotransmitter release. The cytomatrix in the active zone (CAZ) is made up of proteins such as the regulating synaptic membrane exocytosis protein (RIM), RIM-binding proteins (RIM-BPs), ELKS/CAST, Bassoon/Piccolo, Liprin-α, and Munc13-1. RIM is a scaffold protein that interacts with CAZ proteins and presynaptic functional components to affect the docking, priming, and fusion of SVs. RIM is believed to play an important role in regulating the release of neurotransmitters (NTs). In addition, abnormal expression of RIM has been detected in many diseases, such as retinal diseases, Asperger’s syndrome (AS), and degenerative scoliosis. Therefore, we believe that studying the molecular structure of RIM and its role in neurotransmitter release will help to clarify the molecular mechanism of neurotransmitter release and identify targets for the diagnosis and treatment of the aforementioned diseases.

## 1. Introduction

Chemical synapses are an important structure of communication between neurons and are composed of presynaptic terminals, synaptic clefts, and postsynaptic receivers. Neurotransmitters are stored in synaptic vesicles (SVs) (Rizalar et al., 2021). At the presynaptic end, SVs undergo the following processes: activation, transportation, docking, priming, fusion, and exocytosis. Until neurotransmitters are released into the synaptic cleft, electrical signals can be converted into chemical signals and transmitted to the postsynaptic receiver (Sudhof, 2014). The release of neurotransmitters is an extremely complex and delicate process. Many molecules participate in the regulation of this process, including the SNARE complex, Munc18-1, and Ca^2+^ channels (Rizo, 2018). In traditional central synapses, the specific area of the presynaptic end where SVs are released is called the active zone (AZ) (Mochida, 2020). According to electron microscopy, the cytomatrix in the active zone (CAZ) is an electron-dense substance attached to the presynaptic plasma membrane that is composed of a group of insoluble protein complexes anchored in the presynaptic plasma membrane (Sudhof, 2012). This group of protein complexes couples the key components involved in neurotransmitter release in the appropriate regions, such as Ca^2+^ channels and SNARE complexes, so that the vesicle docking, priming, and fusion processes are closely linked (Mochida, 2020). The core members of CAZ are RIM, RIM-BPs, ELKS/CAST, Bassoon/Piccolo, Liprin-α, and Munc13-1 (Südhof T., 2013; Emperador-Melero and Kaeser, 2020).

Among these factors, RIM is an important scaffold protein. Research shows that it closely interacts with other AZ proteins, affects the anchoring and fusion of SVs, regulates the release of neurotransmitters, and can change the plasticity of synapses (Mittelstaedt et al., 2010; Kaeser et al., 2012; Sudhof, 2012).

Additionally, RIM has been found in the pancreas (Kelsell et al., 1998). Pancreas β cells are typical neuroendocrine cells that release insulin in dense core vesicles. RIM plays a regulatory role in insulin release (Yasuda et al., 2010; Park et al., 2012). Moreover, RIM is also detected in the visual and auditory organs (Gebhart et al., 2010; Moser et al., 2020). There is a special synapse called the ribbon synapse in photoreceptor cells and bipolar cells of the retina, vestibular hair cells of the inner ear, and cochlear hair cells. Compared with the conventional central synapses, its structural feature is that CAZ has a special organelle, the synaptic ribbon. The synaptic ribbon is a protein structure that extends into the cytoplasm of the active region and ties a large number of releasable SVs near CAZ (Matthews and Fuchs, 2010). The ribbon synapses of the retina and inner ear transmit light and sound information by releasing neurotransmitters. The calyx of Held in the auditory brainstem is a large synapse. It was found that the effect of the calyx synapse on the recruitment of calcium channels in the RIM knockout mice was significantly weakened (Han et al., 2011). In immature inner ear hair cells of the cochlea, RIM2α is conducive to the stability of Cav1.3 gating dynamics (Gebhart et al., 2010). In terms of vision, Sabrina Mechaussier et al. found a RIM2 mutation in a study of CRSD patients (Mechaussier et al., 2020).

In this article, we will review the molecular structure of members of the RIM protein family, as well as their roles in neurotransmitter release and clinical diseases.

## 2. Characteristics of RIM

In invertebrates, taking *C. elegans* as an example, there is only one gene encoding the RIM protein, named unc10 (Wang and Sudhof, 2003). In vertebrates, RIM is encoded by four genes: *RIM1*, *RIM* 2, *RIM* 3, and *RIM* 4 (Table 1). These genes encode seven RIM isoforms: RIM1α and 1β; RIM2α, 2β and 2γ; RIM3γ; and RIM4γ. The sizes of the human *RIM1* gene, mouse *RIM1* gene, and mouse *RIM2* gene are approximately 500 kb, while that of the human *RIM2* gene is close to 750 kb. Compared with the *RIM1* and *RIM2* genes, the size of the *RIM3* and *RIM4* genes in vertebrates is relatively small. The size of the human *RIM3* gene is close to 15.4 kb, and that of the human *RIM4* gene is close to 54.5 kb (Wang and Sudhof, 2003; Lohner et al., 2017).

**Table 1:** RIM gene and protein names.

Gene name	Gene locus			Protein	
	Mouse	Rat	Human	Protein name	Alternative name
RIM1	1A3	9q13	6q14	RIM1α, RIM1β	RIMS1, RAB3IP2, CORD7, KIAA0340
RIM2	15A2	7q31	8q23	RIM2α, RIM2β, RIM2γ	RIMS2, RAB3IP3, CESDS, OBOE, KIAA0751
RIM3	4D2.2	5q36	1p34.1	RIM3γ	RIMS3, NIM3
RIM4	2H3	3q42	20q13.3	RIM4γ	RIMS4, C20orf190, dJ781B1.3

**Figure 1:**
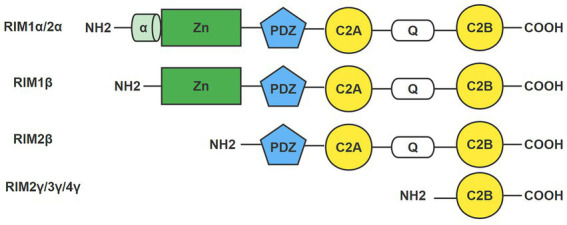
Domain of the RIM. RIM is a multidomain protein with an α helically wrapped zinc finger structure, a PDZ domain, two C2 domains at the C-terminal end, and a proline-rich conservative sequence between the two C2 domains from the N-terminus to the C-terminus. The domains of seven RIM isoforms are shown here: RIM1α/1β, RIM2α/2β/2γ, RIM3γ, and RIM4γ. Only RIM1α and RIM2α include all RIM domains.

RIM is a multidomain protein, and the domains from the N-terminus to the C-terminus are an α helically wrapped zinc finger structure, a PDZ domain, two C2 domains at the C-terminal end, and a proline-rich conserved sequence between the two C2 domains (Figure 1; Mittelstaedt et al., 2010; Kaeser, 2011). It was originally believed that the *RIM1* gene could only be transcribed into RIM1α. However, researchers later found that *RIM1* contains an additional internal promoter that can drive the generation of the RIM1β protein subtype. The RIM 1β protein subtype lacks the N-terminal α-helix (Kaeser et al., 2008). The *RIM2* gene may contain three independent promoters, and RIM2 can be translated into RIM2α, RIM2β and RIM2γ. RIM2β includes all domains except the α spiral regions and zinc finger domains. RIM2γ consists of only a C-terminal C2 domain and a short N-terminal flanking sequence. Among the seven protein subtypes expressed in vertebrates, only RIM1α and RIM2α include all of the RIM domains, which determines the important role of RIM1α and RIM2α in organisms (Mittelstaedt et al., 2010; Kaeser, 2011). The *RIM3* and *RIM4* genes encode RIM3γ and RIM4γ, respectively. They contain the same domains as RIM2γ (Sudhof, 2012).

RIM is expressed in common model organisms such as mice, rats, hamsters, chickens, and cattle. Different RIM protein subtypes are expressed in the cerebrum, hippocampus, cerebellum, calyx synapses, photoreceptors, and spinal cord of rats and mice (Wang et al., 2000; Zanazzi and Matthews, 2009; Gandini and Felix, 2012). RNA preparations from different mouse tissues and brain regions were subjected to Northern blot analyses using cDNA probes specific for RIM1, RIM2, RIM3, or RIM4. RIM1 RNA was found in high concentrations in the brain and at low concentrations in the retina. RIM1 RNA was most prevalent in the cerebrum and cerebellum. RIM2 RNA transcripts were found in the cerebellum at the greatest levels in the brain. RIM3 RNA was found in the cerebrum, cerebellum, brainstem, and olfactory bulb at comparable levels. RIM4 RNA was also found at the greatest concentration in the brain (Uriu et al., 2010). RIM1α and RIM1β are detected in brain area homogenates of wild-type at postnatal day 17. The brain areas detected were the olfactory bulb, ventral striatum, dorsal striatum, hippocampus, frontal cortex, cerebellum, brain stem, and spinal cord (Kaeser et al., 2008).

In particular, Nagase et al. screened and identified a cDNA sequence encoding RIM2 in 1998 and predicted the coding sequence of the corresponding gene, which was named KIAA0751 (Nagase et al., 1998). Subsequently, Mechaussier et al. used three specific primers for RIM2 subtypes to conduct a quantitative analysis of human fetal tissues by PCR technology. The results showed that all transcripts of RIM2 were expressed in the brain, and the expression level was much higher than that in the retina, pancreas, and other tissues. Strong and specific RIM2 immunostaining was observed in the cerebellar cortical neurons (more specifically in Purkinje cells). In the retina, the expression of the three transcripts seems to be similar. In the pancreas, RIM2γ has the highest expression, followed by RIM2α and RIM2β. However, RIM2α is not obviously expressed in fibroblasts, and the main subtype expressed is RIM2γ (Mechaussier et al., 2020). Another study showed that, RIM2 is the prevalent large RIM isoform present at photoreceptor ribbon synapses (Lohner et al., 2017).

Furthermore, RIM was detected in presynaptic nerve terminals close to the active zone and in synaptic ribbons of ribbon synapses of retinal photoreceptor cells (Pernía-Andrade and Jonas, 2011; Lohner et al., 2017; Mochida, 2021).

## 3. RIM and neurotransmitter release

### 3.1. Process of neurotransmitter release

Information transmission between neurons mainly depends on chemical synapses. Neurotransmitters are stored in SVs. SVs undergo a dynamic process of release, recovery, and release at chemical synapses (Gundelfinger et al., 2003). The “classical model of the synaptic vesicle pool” roughly divides the synaptic end vesicles into three subgroups, which are called the reserve pool, recycling pool, and readily releasable pool (RRP) (Rizzoli and Betz, 2005; Denker and Rizzoli, 2010). The vesicles of the RRP are located near the presynaptic membrane and can be released immediately after stimulation. The vesicle capacity of the RRP is limited, but it can change dynamically with the process of release and replenishment (Alabi and Tsien, 2012; Fowler and Staras, 2015). When the action potential reaches the presynaptic end and voltage-gated Ca^2+^ channels (VGCCs) open, the SVs of the RRP fuse with specific areas of the presynaptic membrane, releasing neurotransmitters into the synaptic gap *via* exocytosis and activating postsynaptic receptors (Schoch and Gundelfinger, 2006; Jahn and Fasshauer, 2012).

The CAZ is the location where SVs fuse with the presynaptic plasma membrane (Siksou et al., 2009; Sudhof, 2012; Ackermann et al., 2015). The CAZ of vertebrates is similar to that of invertebrates, with many identical characteristics and core proteins. However, vertebrates have more diverse AZ proteins, which allows them to construct more complex AZs. Morphologically, electron microscopy showed that there were many fine filamentous processes in the CAZ in the central nervous system of vertebrates, which connected the SVs and brought them close to the release site. The sensory synapses in vertebrates are more complex than those in the central nervous system. The vertebrate sensory synapse includes a special organelle, the synaptic ribbon, which connects a large number of SVs near the AZ to promote rapid and sustained transmitter release (Ackermann et al., 2015). The main function of CAZ is to dock vesicles and presynaptic membranes, increase the fusion ability of vesicles, and complete the release of neurotransmitters (Tan et al., 2022). The CAZ is a highly dynamic structure that ensures that vesicle fusion is highly organized in time and space, thus allowing accurate and reliable release of neurotransmitters. Moreover, CAZ regulates presynaptic plasticity (Schoch and Gundelfinger, 2006; Ackermann et al., 2015). In recent years, efforts have been made to identify the molecular components of CAZ. In vertebrate synapses, in addition to SNARES and Sec1/Munc18 (SM) proteins, CAZ also contains Munc13-1, RIMs, RIM-BPs, ELKS/CAST, Bassoon/Piccolo, and Liprin-α (Emperador-Melero and Kaeser, 2020). These protein molecules interact to form a protein network, which is similar to a complex macromolecular structure and operates efficiently during the process of synaptic transmission.

The exocytosis of SVs and presynaptic plasma membranes is strictly regulated and can be divided into three processes: docking, priming, and fusion (Haucke et al., 2011). At present, the “zipper hypothesis” is the most accepted mechanism for the exocytosis of SVs and cell membranes. It involves an important structure, the SNARE complex, which is composed of three proteins. On the synaptic vesicle, these three proteins are synaptobrevin 2 (Syb2, also known as VAMP2), and on the presynaptic membrane, they are SNAP-25 and Syntaxin-1. According to the SNARE protein distribution, researchers named Synatobrevin2 and its related proteins located in the vesicle membrane vascular SNARE (v-SNARE). Syntaxin-1 and SNAP-25 located in the presynaptic membrane are called target SNARE (t-SNARE). The SNARE complex is a molecular machine that mediates the fusion of SVs and presynaptic membranes (Li et al., 2018; Wang et al., 2019). In the SNARE complex, Synatopbrevin2 and Syntaxin-1 provide α spirals, and SNAP-25 provides two α spirals, forming 4 α helical bundles. Such structures shorten the distance between vesicles and cell membranes. The SNARE complex provides power for membrane fusion. When the action potential reaches the end of the synapse, calcium ions flow in, and the SNARE complex moves to both sides like a zipper, leading to the formation of fusion pores between the vesicle and the presynaptic membrane. Subsequently, neurotransmitters are released through fusion pores (Li et al., 2018; Rizo, 2018; Wang et al., 2019).

### 3.2. RIM is involved in neurotransmitter release

The proteins in the AZ play a crucial role in the release of neurotransmitters. These proteins recruit calcium channels and key components involved in the release of vesicles into appropriate regions, thus facilitating the docking, priming, and fusion of vesicles (Sudhof, 2012). Among them, RIM plays a very important role.

1. RIM may be the key protein controlling the CAZ protein network. The multidomain structure of RIM interacts indirectly or directly with other AZ proteins and other presynaptic functional components and acts as a protein scaffold in the AZ (Schoch et al., 2002; Mittelstaedt et al., 2010; Tan et al., 2022).
2. RIM affects the release of SVs, especially during the process of vesicle priming and fusion. The RIM protein collects SVs and the Munc13-1 protein at the presynaptic membrane, catalyzes the formation of the SNARE complex, and increases the fusion ability of vesicles (Sudhof T. C., 2013; Südhof T. C., 2013). In addition, RIMs and RIM-BPs jointly anchor calcium channels to release sites. In 2019, researchers reported that the AZ proteins RIM and RIM-binding proteins (RIM-BPs) can be separated, and VGCCs can be gathered to form a functional area for neurotransmitter release in CAZ (Wu et al., 2019).
3. RIM can affect synaptic plasticity. Synaptic plasticity refers to the adjustable strength of synaptic connections. Synaptic plasticity can be divided into short-term synaptic plasticity and long-term synaptic plasticity (Barroso-Flores et al., 2017). Long-term plasticity includes long-term potentiation (LTP) and long-term depression (LTD) (Michmizos et al., 2011). Research has shown that in different types of synapses, RIM1α is essential for different forms of synaptic plasticity (Castillo et al., 2002; Kaeser and Sudhof, 2005; Kintscher et al., 2013).

## 4. Interactions between RIM and presynaptic terminal molecules

**Figure 2:**
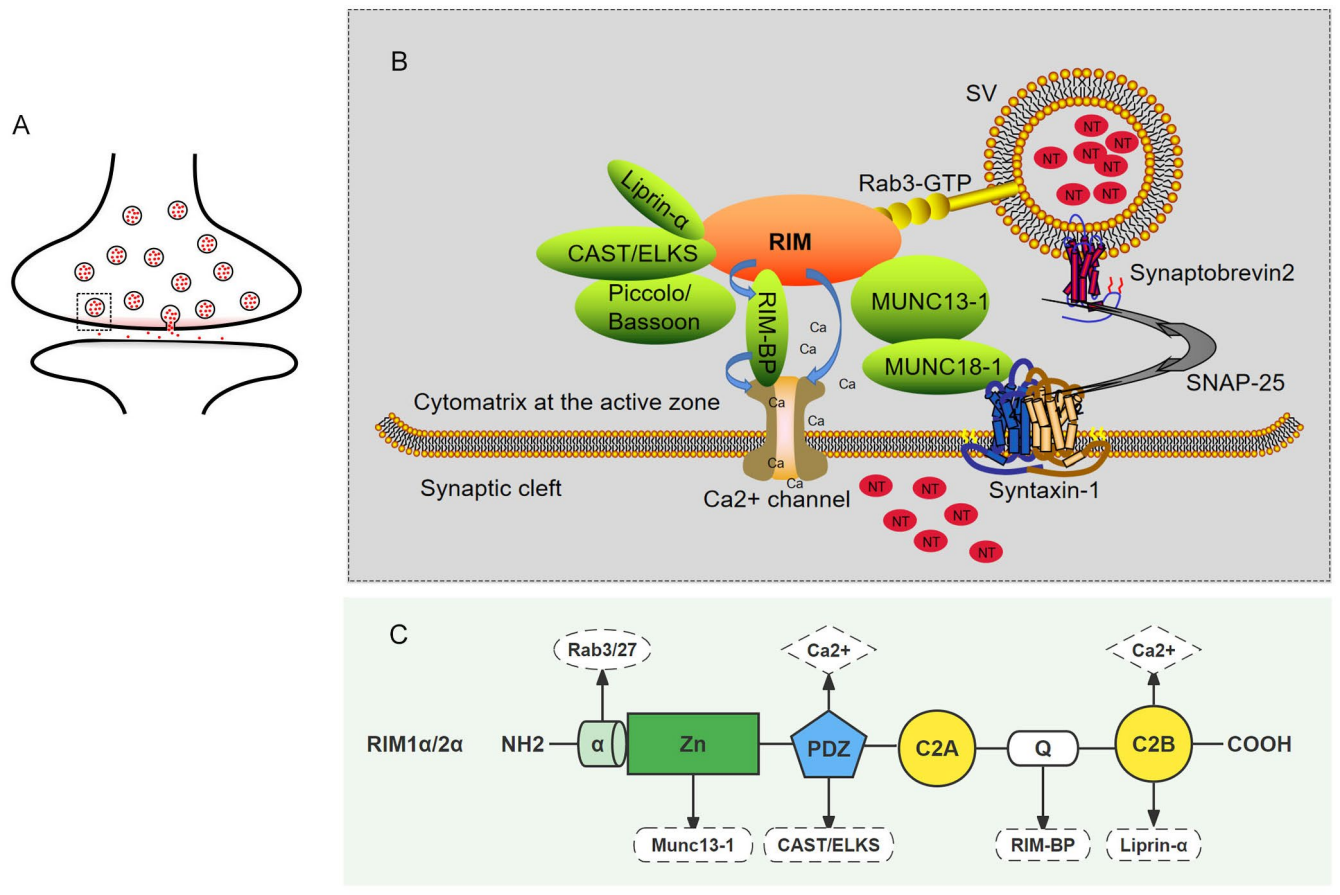
The role of RIM proteins in CAZ. **(A)** Schematic of a synapse. The small circle represents synaptic vesicles, the small red dots contained in the circle represent neurotransmitters, and the gray rectangle (dotted line) represents an active zone. **(B)** A magnified view of the active zone from the gray rectangle (dotted line) in A. in which interactions between RIM and presynaptic terminal molecules are shown. The upper part is the active zone, the middle part is the presynaptic membrane, and the lower part is the synaptic cleft. The functional elements of the neurotransmitter release mechanism are shown: vesicular protein Rab3 (yellow) protein molecules in the active region (green); the snare proteins VAMP, syntaxin-1, and SNAP-25; the SM proteins Munc18-1 and Munc13-1 as well as calcium channels on the presynaptic membrane; and calcium ions. In the active zone, RIM acts as a scaffold to position important elements at appropriate locations in the active region. **(C)** The sites of the interactions between the RIM domain and important elements required for neurotransmitter release.

**Figure 3:**
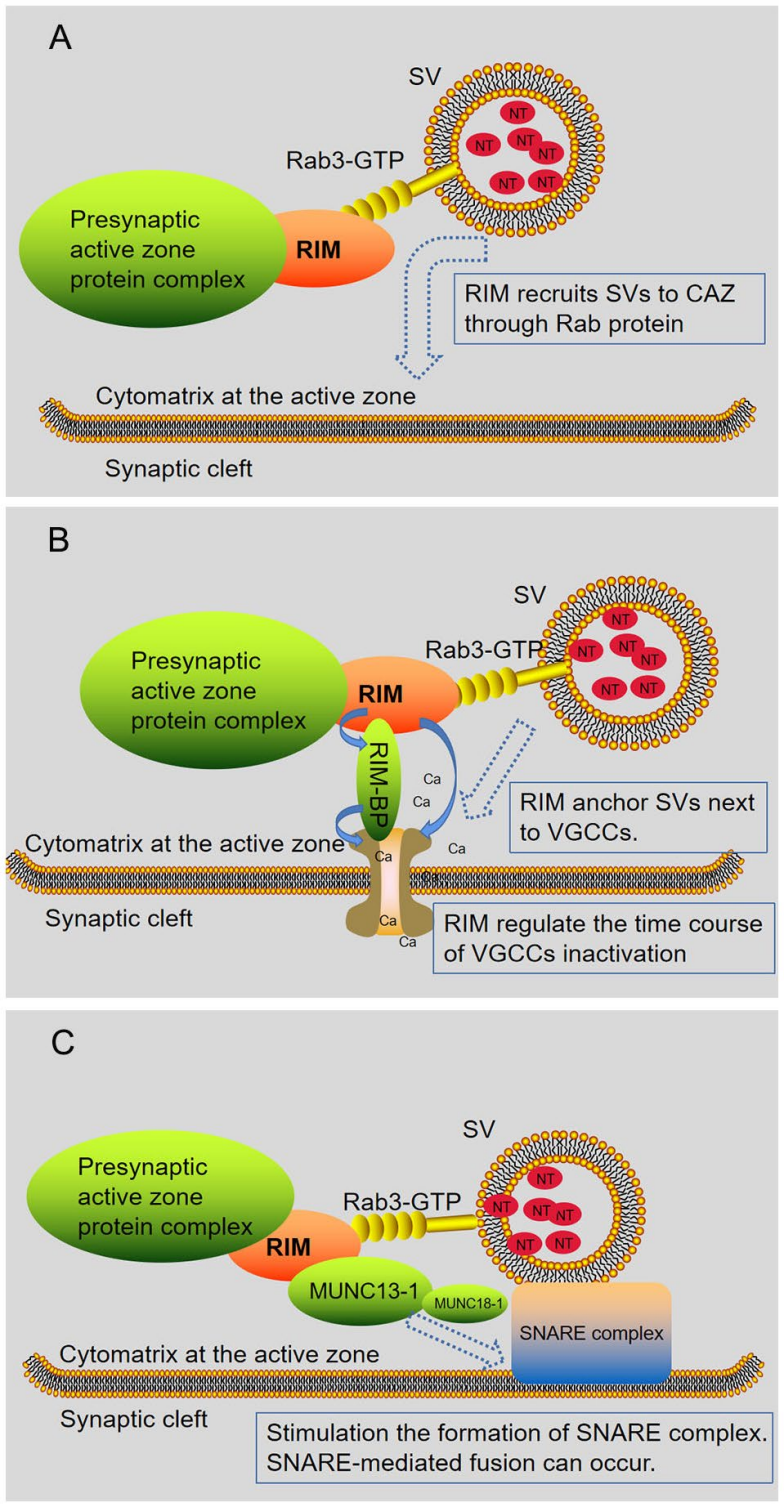
RIM regulates the release of neurotransmitters. RIM affects specific stages of the SV exocytotic cycle. **(A)** RIM recruits SV *via* Rab proteins (Rab3 and Rab27 subtypes). **(B)** RIM anchors SV next to VGCCs. RIM directly binds Ca2 + channels or indirectly binds them through RIM-BPs, recruiting Ca2 + channels for rapid excitation-secretion coupling. In addition, RIM regulates the time course of VGCC inactivation, resulting in a sustained Ca2+ influx. **(C)** RIM, Rab3, and Munc13-1 regulate the late steps in SV fusion. Subsequently, the Munc13-MUN domain activated the Munc18-1/Syx1 complex, thus promoting the formation of the SNARE complex. After this step, an action potential is triggered, and SNARE-mediated fusion can occur. In short, RIM affects the docking of SV with fusion mechanism proteins (SNARE) and Ca2+ channels.

According to research, RIM mainly interacts with the following categories of molecules at the presynaptic end: synaptic vesicle proteins (such as Rab), ion channels of presynaptic membranes (voltage gated calcium channels, VGCCs), and protein molecules of CAZ (such as Munc13, RIM-BP, ELKS/CAST, Liprin-α, and Bassoon/Piccolo) (Table 2; Pavlos and Jahn, 2011; Dolphin and Lee, 2020; Mochida, 2020). Next, we will explain how RIM interacts with the above components (Figure 2) and the impact of their interactions on the release of neurotransmitters (Figure 3).

**Table 2:** Important elements in the active zone that interact with RIM.

Protein family	Isoforms interacting with RIM	Functions in the CAZ	Sites of Interaction with RIM	References
Rab	Rab3/27B	Transport synaptic vesicles to CAZ	Rab3A-GTP binds to the α Helix (next to the zinc finger domain of RIM). The binding site of Rab27B is unknown.	Dulubova et al. (2005), Pavlos and Jahn (2011), Shin (2014)
VGCCs	Cav2.1/2.2/2.3	Calcium influx triggers rapid neurotransmitter release	The PDZ domain of RIM binds with the C-terminus of N - and P/Q-type Ca^2+^channels to recruit calcium channels. RIM C2B domain binding to Ca^2+^channel β subunits inhibits the inactivation of VGCCs.	Kiyonaka et al. (2007), Kaeser et al. (2011, 2012), Gandini and Felix (2012)
Munc13	Munc13-1	Mediate synaptic vesicle docking and priming	The C2A domain of Munc13 binds with the ZF domain of the RIM protein to form the Munc13-RIM heterodimer.	Andrews-Zwilling et al. (2006), Deng et al. (2011), Camacho et al. (2017), Zarebidaki et al. (2020)
RIM-BP	RIM-BP1/2	Stabilize the structure of CAZ; recruit calcium channels	The SH3 domain of RIM-BP can bind to a PXXP motif between the two C2 domains of the RIM protein.	Acuna et al. (2015), Wu et al. (2019), Gao et al. (2021)
ELKS/CAST	ELKS1αB/2αB	As a molecular hub, it interacts with other CAZ components to support the release of neurotransmitters	The amino acid motif IWA at the end of the fourth CC domain at the C-terminus of ELKS1αB/2αB was found to bind to the PDZ domain of RIM1.	Ohtsuka et al. (2002), Lu et al. (2005)
Liprin-α	Liprin-α1/2/3/4	Participate in the CAZ assembly	The curly spiral region of Liprin-α binds to CAST, and the same Liprin-α region also interacts with RIM’s C2B domain.	Schoch et al. (2002), Ko et al. (2003), Spangler and Hoogenraad (2007)
Bassoon/Piccolo	Bassoon/Piccolo	Participate in the CAZ assembly	RIM1 and Piccolo/Bassoon bind directly to the carboxyl end and central region of CAST, respectively	Takao-Rikitsu et al. (2004)

### 4.1. Interaction between RIM and Rab

The Rab protein is a GTP-binding protein with a small molecule. It is widely expressed in cells and is present in organelle membranes and plasma membranes (Regazzi, 2008). All Rab proteins have a common G domain, including six β folds, five α helices, and five polypeptide rings. Rab protein switches between active Rab-GTP and inactive Rab-GDP, which is regulated by guanine nucleotide exchange factors (GEFs) and GTPase-activating proteins (GAPs) (Regazzi, 2008; Stein et al., 2012). During vesicle transport, the Rab protein is involved in budding to form vesicles, regulating vesicle movement along the cytoskeleton, and anchoring and fusion of vesicles (Hutagalung and Novick, 2011). In the Rab protein family, at least 11 different Rab proteins coexist on SVs (Pavlos and Jahn, 2011).

It has been found that the Rab3 and Rab27 subfamilies function in regulating the exocytosis of SVs (Tsuboi and Fukuda, 2006; Mignogna and D'Adamo, 2018). Rab3 is encoded by four genes, which drive the expression of Rab3a, b, c, and d. Among these proteins, Rab3a, Rab3b, and Rab3c are expressed in SVs, and Rab3a is the most abundant subtype. Rab3d is mainly expressed in the pancreas, mast cells, and parotid glands (Shin, 2014). Rab3a is attached to SVs by C-terminal targeting. As a synaptic vesicle protein, Rab3a plays an important role in the exocytosis of SVs and acts as a regulatory factor for neurotransmitter release (Schlüter et al., 2004; Shin, 2014).

The Rab3a protein interacts with its target protein in a GTP-dependent manner. The first known Rab3a effector was RIM. Initially, researchers found that GTP-Rab3a interacted with the effector protein RIM, which enhanced exocytosis (Wang et al., 1997). The initial study concluded that RIM passed the α helix and interacted with Rab3a. Here, the α helix is located next to the RIM zinc finger domain. Rab3a simultaneously binds to the α helix and a SGAWFF motif at the end of the short α helix (Betz et al., 2001; Wang et al., 2001). Later, Dulubova et al. showed by using nuclear magnetic resonance spectroscopy that Munc13-1, RIM, and GTP-Rab3a can form a complex. The binding sites of Rab3a and Munc13-1 to RIM are adjacent but separate, and the binding sites for these proteins are located in different regions of the N-terminal sequence of RIM. The binding of Munc13-1 and RIM changes the binding modes of RIM and Rab3. When RIM binds Rab3a, the SGAWFF motif is no longer needed, and the SGAWFY motif is released. This allows the formation of a Rab3a/RIM/Munc13-1 ternary complex. The above results show that the N-terminal sequence of RIM has a modular design, which can place Rab3a and Munc13-1 in a close but not competitive position (Dulubova et al., 2005). The formation of the RIM/Munc13/Rab3a complex is considered a key component of synaptic vesicle docking. RIM recruit vesicles through Rab3 and activates Munc13-1. This interaction may ensure that SVs are appropriately targeted to the AZ of the plasma membrane (Figure 3A). The selective disruption of this interaction in the calyx synapse will reduce the size of the RRP (Sakane et al., 2006; Sudhof, 2012). Except for RIM1α/2α, other members of the RIM protein family lack the domain required for Rab3 binding, so their role in exocytosis is unclear. However, if they play a role in exocytosis, they are probably not related to the Rab3 GTPase (Kaeser et al., 2008; Regazzi, 2008).

Rab27 is also considered to be a regulator of exocytosis. Only Rab27b, not Rab27a, was expressed in SVs isolated from the rat brain (Hutagalung and Novick, 2011). Rab27b partially overlaps with Rab3 at synaptic nerve endings, and it is closely related to Rab3 in structure, indicating that Rab27b may be involved in the exocytosis of SVs (Fukuda, 2003; Schlüter et al., 2004; Pavlos et al., 2010). Similar to Rab3a, Rab27b may regulate the activation of SVs in CAZ by interacting with RIM and Munc13 in a GTP-dependent manner. However, the physiological function of the interaction between rab27b and other molecules at synaptic terminals is still unclear.

### 4.2. Interaction between RIM and VGCCs

Voltage-gated Ca^2+^ channels (VGCCs) are the main pathways of Ca^2+^ influx at axon terminals, and they are crucial for neurotransmitter release. VGCCs can be divided into low-voltage-activated Ca^2+^ channels (LVAs) and high-voltage-activated Ca^2+^ channels (HVAs) according to the threshold potential of activated ion channels (Simms and Zamponi, 2014). Researchers divided VGCCs into L-type, N-type, and T-type channels according to their sensitivity to changes in neuronal membrane potential. Subsequently, P/Q-type and N-type VGCCs were also found in neurons. VGCCs are made up of four or five different subunits, which include α1, α2, δ, β, and γ (Catterall, 2011). Moreover, there are many coding genes for each subunit, resulting in a diversity of Ca^2+^ channels at the molecular level. Recent findings show that the α1 subunit is encoded by 10 different genes. The γ subunits are mainly found in skeletal muscle but not in the brain. Under normal conditions, a α2 subunit and a δ subunit form a α2δ complex. In the mammalian nervous system, VGCCs are mainly composed of three parts: α1, α2δ, and β. Subunit α1 is the main component of the formation of Ca^2+^ channels. P/Q-type Ca^2+^ channels are composed of the α1A subunit (Cav 2.1), N-type Ca^2+^ channels consist of the α1B subunit (Cav2. 2), and the R-type Ca^2+^ channels consist of the α1E subunit (Cav2.3). The release of neurotransmitters almost entirely depends on N-type and P/Q-type Ca^2+^ channels, but R-type Ca^2+^ channels may also contribute (Zamponi, 2003; Simms and Zamponi, 2014; Mochida, 2018; Dolphin and Lee, 2020). The conventional central synapses and ribbon synapses differ in that the latter relies primarily on L-type Ca^2+^ channels for release, while the former relies more on N-and/or P/q-type Ca^2+^ channels (Catterall, 2011).

The location and number of Ca^2+^channels at synaptic terminals, as well as the change in calcium channel function, will affect the release of neurotransmitters. At chemical synapses, action potentials trigger Ca^2+^ influx and then promote the release of neurotransmitters, completing the conversion of electrical signals to chemical signals (Kaeser et al., 2011; Simms and Zamponi, 2014). Various factors regulate the anchoring and function of VGCCs at synaptic terminals (Gandini and Felix, 2012; Wu et al., 2019). RIM plays an important role in the localization and functional regulation of VGCCs. Here, we will review the latest progress in research on the interaction between VGCCs and RIM.

The important function of RIM is to recruit VGCCs. By generating conditional knockout mice lacking all multidomain RIM subtypes and using electrophysiological records and quantitative immunostaining of Ca^2+^ channels, researchers found that the loss of the RIM protein disrupted the initiation of SVs, reduced the presynaptic localization of Ca^2+^ channels, and eliminated the release of most neurotransmitters. This shows that RIMs are crucial for localizing Ca^2+^ channels to release sites (Kaeser et al., 2011). Similarly, by using conditional knockout mice, the study showed that the acute single deletion of a single RIM gene reduced release and vesicle priming but did not change extracellular Ca^2+^ influx and did not affect the synchronization of vesicle release. In contrast, the deletion of RIM1/2 genes seriously impaired the synchronization of Ca^2+^ influx and vesicle release. This indicates that RIM proteins of different subtypes compensate for each other when recruiting Ca^2+^ channels to the AZ (Kaeser et al., 2012). Furthermore, a different study demonstrates that RIM proteins are crucial for luring Ca^2+^ channels to the AZ. The results showed that the Ca^2+^ current density of chemical synapses was significantly reduced (by approximately twofold) in the absence of all RIM1/2 subtypes, and the number of vesicles near the AZ was reduced (Han et al., 2011).

Research shows that RIMs affect VGCCs’ function from two perspectives (Figure 3B). On the one hand, RIMs affect the number of Ca^2+^ channels in the AZ. RIM recruited Ca^2+^ channels to the AZ in two ways, thus shortening the distance between vesicles and calcium channels. The first path involves the direct interaction between the PDM domain of RIM and the C-terminus of presynaptic N-and P/Q-type Ca^2+^ channels (Kaeser et al., 2011; Sudhof, 2012). The second path is the binding of RIM PxxP sequences with RIM-BP and the direct binding of RIM-BP to the C-terminus of Ca^2+^ channels (Kaeser et al., 2012). Recently, Wu et al. showed that the presynaptic AZ scaffold proteins RIM and RIM-BP form self-assembled condensates *via* liquid–liquid phase separations capable of clustering voltage-gated Ca^2+^ channels on lipid membrane bilayers (Wu et al., 2019). In mice lacking RIM and/or RIM-BP, the density of Cav2.1 channels at synaptic terminals decreased (Dolphin and Lee, 2020). On the other hand, RIMs regulate the VGCCs mechanism of action. The C2B domain of RIM binds the β subunit, thereby inhibiting the inactivation of VGCCs and indirectly increasing the release of neurotransmitters (Kiyonaka et al., 2007). This regulatory effect is not limited to α-RIMs, and research shows that γ-RIMs can also bind with β subunits to significantly inhibit the inactivation of VDCCs (Uriu et al., 2010).

In addition to the central nervous system, synaptic studies in the visual and auditory systems have also shown that RIM regulates Ca^2+^ channels in synaptic terminals. For example, RIM2α and RIM2β promote the abundance of voltage-gated CaV1.3 in the AZ of hair cells, which is necessary for normal hearing (Jung et al., 2015). Similarly, Tobias Moser et al. found that the C2B domains of RIM2α and RIM3γ interact with the c-terminal of the pore-forming α subunit of the CaV1.3 channel, which mediates the stimulation-secretion coupling of banded synapses in cochlear inner hair cells (IHC) (Picher et al., 2017). RIM1/2 is an important regulator of Cav1.4 channel function in mouse rod photoreceptors. Opening of the Cav1.4 channel mediated by RIM1/2 is necessary in mouse rod photoreceptors. In particular, in rod photoreceptors, RIM1/2 did not affect the recruitment of Cav1.4 channels to the synaptic zone but significantly enhanced the opening of these channels. Therefore, the effects of RIM on the release of Ca^2+^ channels and neurotransmitters may vary depending on the subtype of VGCCs and the type of synapse (Grabner et al., 2015; Mochida, 2018).

### 4.3. Interaction between RIM and Munc13

Munc13 is the homolog of *C. elegans* Unc-13 and *Drosophila* Dunc-13 in mammals. Four subtypes of Munc13 have been found in mammals, namely Munc13-1, 2, 3, and 4 (Koch et al., 2000). Munc13-1 and Munc13-3 are specifically expressed in the nervous system and neuroendocrine cells. Munc13-1 and Munc13-3 are more localized at presynaptic terminals. Munc13-1 is very abundant in CAZ, which suggests that it plays a role in the release of neurotransmitters (Song et al., 1998). Munc13-1 is a complex multidomain protein. In addition to the C1 domain, it also contains three C2 domains and MUN structures. The MUN domain, which is “banana”-shaped according to physics data and approximately 15 nm long, is divided into four subdomains (Yang et al., 2015; Li et al., 2021).

Munc13-1 interacts with a variety of molecules through its multiple domains, such as syntaxin-1, Munc18, and RIM. These molecules are related to the release of SVs, which indicates that Munc13-1 plays a regulatory role in the release of SVs (Wang et al., 2020). The main role of Munc13-1 in the neurotransmitter release process is to promote the assembly of SNARE complexes and participate in regulating the docking and fusion of SVs (Li et al., 2021). The SNARE complex is a molecular machine that mediates the fusion of SVs and presynaptic membranes. The key molecules of SNARE complex assembly include Syntaxin-1, SNAP-25, and VAMP2, and this process is regulated by Munc13-1 and Munc18-1. Syntaxin-1 has four α-spirals, three α-helices that form the Habc domain, and α-helices that form the H3 domain. First, Munc18-1 and Syntaxin-1 bind to form the Munc18-Syntaxin complex. The Habc domain of Syntaxin-1 binds with the H3 domain that participates in the formation of the SNARE complex, so Syntaxin-1 is closed and cannot participate in the formation of the SNARE complex. However, the central MUN domain of Munc13-1 can bind with Syntaxin-1 to release the H3 domain from the Munc18-Syntaxin complex, thus bringing Syntaxin-1, SNAP-25, and VAMP2 together to form a SNARE complex to promote vesicle and membrane fusion (Wang et al., 2020; Li et al., 2021). In addition, some studies have shown that the Munc13-1 MUN domain can interact with VAMP2 to promote the assembly and membrane fusion of the SNARE complex (Wang et al., 2019).

It is discovered that RIM and Munc13-1 contribute to the localization and release of SVs in positive and largely independent ways. On the one hand, RIM can independently regulate the location of SVs near the AZ membrane and does not depend on Munc13-1. On the other hand, RIM and Munc13-1 both mediate synaptic vesicle docking and priming (Figure 3C), and RIM can activate Munc13-1, which largely proves that RIM acts upstream of Munc13-1 in SV docking. The concentration dependence of Munc13-1 initiation was tested, and the results showed that RIM improved Munc13-1 initiation function by approximately fourfold (Zarebidaki et al., 2020). In addition, the AZ recruitment of the Munc13-1 and ubMunc13-2 subtypes of Munc13 is regulated by RIM1 (Andrews-Zwilling et al., 2006). Although the RIM and Munc13-1 connection plays an obvious function in vesicle docking and release, there may be other mechanisms that allow SVs to fuse (Zarebidaki et al., 2020).

One study found that the C2A domain of Munc13-1 has a positive effect, interacting with the zinc finger domain of the RIM protein to promote vesicle docking, priming, and fusion. In terms of mechanism, it is believed that the function of vesicle initiation is inhibited due to the homodimerization of the C2A domain of Munc13-1, and RIM disrupts the homodimerization of the C2A domain of Munc13-1. Only when Munc13-1 is heterodimerized with RIM through the C2A domain can vesicles effectively complete docking and priming. This shows that the Munc13-RIM heterodimer is the active component for vesicle docking and initiation (Deng et al., 2011; Rizo, 2018; Liu et al., 2019). According to the previous description, RIM contains adjacent but separated Munc13-1 and Rab3 binding sites, allowing the formation of a three-part Rab3/RIM/Munc13-1 complex. The formation of this complex may directly regulate the release probability of neurotransmitters (Dulubova et al., 2005; Rizo, 2018).

### 4.4. Interaction between RIM and RIM-BPs

RIM-BPs are a class of multidomain proteins that include three SH3 domains, two or three adjacent fibronectin type III domains (FNIII), and N-terminal subtype-specific regions (Mittelstaedt and Schoch, 2007). There are three genes encoding RIM-BPs, namely *RIM-BP1*, *RIM-BP2*, and *RIM-BP3*. The vertebrate genome contains at least two *RIM-BP* genes, and the *RIMBP3* gene exists only in mammals. Research shows that RIM-BP1 and RIM-BP2 are both expressed in the mouse brain, while RIM-BP3 is mostly detected at high levels outside the nervous system (Mittelstaedt and Schoch, 2007).RIM-BP, an important CAZ protein, plays an important role in stabilizing the CAZ structure and recruiting calcium channels (Emperador-Melero and Kaeser, 2019; Gao et al., 2021). Tobias Moser et al. found that RIM-BP1/2 is required for normal sound encoding at the synapses of inner ear hair cells. RIM-BP2 seems to play a dominant role (Krinner et al., 2021). RIM-BP2 positively regulates the number of CaV1.3 synaptic channels, thereby promoting the release of synaptic vesicles (Stefanie et al., 2017).

RIM-BPs were identified as the binding partners of the presynaptic AZ proteins RIMs and VGCCs. RIM-BPs can localize VGCCs to the presynaptic membrane through their SH3 domain, reducing the distance between vesicles and channels to the nanometer scale (Luo et al., 2017). This specific distance is necessary to ensure the high fidelity of vesicle release and the accuracy of information transmission between neurons. RIM-BPs can bind three types of channels (N-, P/Q-, and L-type Ca^2+^ channels) (Acuna et al., 2015). In addition, the SH3 domain of RIM-BPs can also bind with the PXXP motif between the two C2 domains of RIM. Then, RIMs indirectly link N-and P/Q-type Ca^2+^ channels to the presynaptic AZ through the PDZ domain and build a stable triangular structure so that calcium channels are firmly tethered to the presynaptic membrane (Hibino et al., 2002; Kaeser et al., 2011; Acuna et al., 2015; Li and Kavalali, 2015; Gao et al., 2021). Knockout of *RIM-BP2* in hippocampal neurons can cause changes in the location of calcium channels in presynaptic membranes, affect the coupling distance between calcium channels and release sites, and reduce the probability of vesicle release (Grauel et al., 2016). Depletion of Ca^2+^ channels in ribbon synapses was observed due to RIM-BP deletion but not in the calyx of Held. This suggests that the L-type Ca^2+^ channels of banded synapses mainly rely on RIM-BPs for localization, while the N-type and P/Q-type Ca^2+^ channels found in standard synapses (such as the calyx of Held) depend on both RIM-BPs and RIM (Luo et al., 2017). As mentioned above, Wu et al. showed that the presynaptic AZ scaffold proteins RIM and RIM-BP form self-assembled condensates *via* liquid–liquid phase separations capable of clustering voltage-gated Ca^2+^ channels on lipid membrane bilayers (Wu et al., 2019). Functional redundancy exists between RIM-BP and RIM (Kushibiki et al., 2019). Studies have shown that the simultaneous loss of RIM-BP and RIM seriously affects the recruitment of Ca^2+^ channels and vesicle anchoring (Acuna et al., 2016).

### 4.5. Interaction between RIM and ELKS/CAST

The ELKS/CAST protein family is also an important component of the AZ (Hida and Ohtsuka, 2010). The vertebrate genome expresses two ELKS genes, *Erc1* and *Erc2*, which produce ELKS1 and ELKS2 proteins. Humans express two Erc genes, and their protein products have sequence homology of approximately 99% with their mouse homologs. ELKS is named because its protein is rich in glutamate (E), leucine (L), lysine (K), and serine (S). ELKS has at least five alternative splicing subtypes: ELKSα, ELKSβ, ELKSγ, ELKSδ, and ELKSε. ELKSα is mainly expressed in the brain, is divided into the ELKS1αB and ELKS2αB subtypes, and has high homology with CAST at the protein level (Monier et al., 2002; Ohtsuka et al., 2002; Hida and Ohtsuka, 2010). CAST and ELKSα are both 120 kDa proteins with 4 CC regions (CCA/CCB/CCC/CCD) and a unique C-terminal amino acid motif, IWA (Held and Kaeser, 2018). In the mouse hippocampus, CAST is localized near the presynaptic plasma membrane. Similar to CAST, ELKSα is localized near the presynaptic plasma membrane in the mouse cerebellum (Ohtsuka et al., 2002; Deguchi-Tawarada et al., 2004).

CAST/ELKS affects the release of neurotransmitters (Held and Kaeser, 2018). One study showed that the amino acid motif IWA at the end of the fourth CC domain at the C-terminus of ELKS1αB/2αB was required for specific interactions with the PDZ domain of RIM1 (Ohtsuka et al., 2002; Lu et al., 2005). Furthermore, ELKS binds to the presynaptic Liprin-α protein *via* the N-terminal helix–helix region (CCA-CCC) (Ko et al., 2003). The central curvilinear circle (CCB and CCC domains) binds to the AZ components Bassoon and Piccolo (Takao-Rikitsu et al., 2004). The C-terminal CCD domain interacts with the β subunits of voltage-gated Ca2+ channels (CaVβ), specifically CaVβ4 (Billings et al., 2012). The C-terminus of ELKS1 interacts specifically with the N-terminal region of bMunc13-2 (Kawabe et al., 2017). In this way, a protein–protein interaction network is formed in CAZ.

ELKS/CAST family members are scaffold protein molecules in the AZ, the destruction of which impairs the fusion of SVs. Knocking out all ELKS and RIM proteins at the same time leads to the breakdown of the AZ, accompanied by an almost complete loss of docked SVs (Wang et al., 2016; Held and Kaeser, 2018). Disruption of the CAST-RIM interaction results in incorrect localization of RIM1 in the AZ but has no effect on the localization of CAST, suggesting that CAST may act as an anchor protein for RIM1 in the presynaptic AZ (Ohtsuka et al., 2002). In CAST/ERC2 knockout mice, the level of soluble RIM1 was slightly elevated. Therefore, CAST may regulate the homeostasis of RIM1 in the presynaptic AZ (Hamada and Ohtsuka, 2018).

### 4.6. Interaction between RIM and Liprin-α

The Liprin-α family contains 4 members in vertebrates (Liprin-α1/2/3/4) and one member each in *C. elegans* and *Drosophila*, named SYD-2 and Dliprin-α, respectively (Serra-Pagès et al., 1998; Kaufmann et al., 2002; Astigarraga et al., 2010). In mammals, Liprin-α1 is widely expressed, while Liprin-α2/3 is mainly expressed in the brain, and Liprin-α4 is expressed in both the brain and testes (Zürner and Schoch, 2009; Wong et al., 2018). Liprin-α consists of three tandem SAM (Seart-α-motif) domains (SAM1/2/3) at the C-terminus and N-terminal helixes, according to sequence analysis (Spangler and Hoogenraad, 2007).

The liprin-α family is also involved in the construction of CAZs and the release of neurotransmitters. To begin, the curly spiral region of liprin-α binds to CAST and then interacts with the RIM C2B domain to form a complex. Then, RIM binds to Rab3-GTP, which, in turn, indirectly affects the activity of SVs (Zürner and Schoch, 2009; Wong et al., 2018; Xie et al., 2021).

### 4.7. Interaction between RIM and Bassoon/Piccolo

Bassoon/Piccolo are large (>400 kDa) multidomain proteins of CAZ that are present in synapses in all vertebrates. Bassoon/Piccolo consists of highly homologous zinc finger and coiled-coil circle (CC) sequences (predicted coiled-coil domains). The difference is that Piccolo contains an N-terminal glutamine-rich sequence, a C-terminal PDZ domain, and two C-terminal C2 domains (C2A and C2B) (Fenster et al., 2000; Garcia et al., 2004; Gundelfinger et al., 2015).

Bassoon/Piccolo have highly similar modular structures with overlapping functions. Mukherjee et al. (2010) demonstrated through gene knockout that Piccolo and Bassoon tubes play a redundant role in synaptic vesicle aggregation at nerve endings without directly participating in the release of neurotransmitters (Mukherjee et al., 2010). Piccolo/Bassoon do not interact directly with RIM. The CAST protein family was discovered to be indirectly related to Piccolo/Bassoon. RIM1 and Piccolo/Bassoon bind directly to the carboxyl end and central region of CAST, respectively, and participate in the release of neurotransmitters (Takao-Rikitsu et al., 2004).

## 5. RIM and synaptic plasticity

Synaptic plasticity refers to the ability to modify the strength of synaptic connections. Long-term and short-term synaptic plasticity are two categories of synaptic plasticity. Nowadays, the role of synaptic plasticity in learning and memory is gradually being recognized. RIM1a knockout mice show significant impairment in learning and memory (Powell et al., 2004).

Research has revealed RIM1 to be crucial for both long-and short-term synaptic plasticity in several types of synapses (Castillo et al., 2002; Kaeser and Sudhof, 2005; Kintscher et al., 2013). In CA1-region Schaffer collateral excitatory synapses and in GABAergic synapses, RIM1α is required for maintaining normal neurotransmitter release and short-term synaptic plasticity. In contrast, PKA-dependent presynaptic long-term plasticity in mossy fiber synapses is abolished in RIM1α and in Rab3A KO mice, but the short-term synaptic plasticity appears to be unchanged (Castillo et al., 2002; Kaeser and Sudhof, 2005). Another study found that RIM1a can affect the short-term plasticity of cerebellar parallel fiber synapses (Kintscher et al., 2013).

However, the molecular mechanism by which RIM affects synaptic plasticity has not been clarified. It was proposed that subtype 7 metabotropic glutamate receptor (mGluR7) and RIM1a interact to control LTP at the hippocampal mossy fiber stratum lucidum interneuron (MF-SLIN) synapses. Internalization of mGluR7 would release RIM1a or its substrate, making the MF-interneuron terminals LTP competent (Pelkey and Mcbain, 2008). Moreover, the cAMP/PKA signaling pathway is thought to be associated with synaptic plasticity (Huang et al., 2005; Chevaleyre et al., 2007; Mittelstaedt et al., 2010). But it is still unclear exactly how it works.

## 6. RIMs and disease

### 6.1. Cone-rod dystrophy 7

Cone-rod dystrophy 7 (CORD7) is an autosomal dominant cone rod dystrophy linked to chromosome 6q14 (Kelsell et al., 1998). The disease is characterized by retinal pigment deposition, which is mainly located in the macular region, while cone photoreceptors are reduced and rod degeneration occurs. The main symptoms of patients are decreased vision and sensitivity of the central visual field, followed by peripheral vision loss (Kelsell et al., 1998). It was mentioned earlier, RIM is expressed in the ribbon synapses of the visual system (Lohner et al., 2017; Moser et al., 2020). A study found that *RIM1* may be a candidate gene for causing the disease. The C2A domain gene mutation of RIM1 causes the C2A domain to be replaced, which may change the affinity of RIM1 for the L-type Ca^2+^ channel, thus changing the rate of neurotransmitter release from vesicles. This change in neurotransmitter release may have a long-term impact on the viability of photoreceptors. However, it is necessary to identify *RIM1* mutations in other CORD7 patients or prove the functional defect of the RIM1 protein with a mutation at this site to confirm that *RIM1* mutations truly cause this disease (Johnson et al., 2003).

### 6.2. Congenital nonprogressive cone-rod synaptic disorder syndrome

CRSDS is a genetic retinal disease characterized by retinal and neurodevelopmental diseases, as well as abnormal glucose homeostasis. The main clinical manifestations in patients are poor vision, photophobia, and nystagmus, and the light adaptation response is severely weakened and delayed (Mechaussier et al., 2020).

In photoreceptor synapses, RIM2 plays an important role in normal synaptic connections (Moser et al., 2020). Loss of RIM2 function leads to syndromic congenital cone rod synapsis with neurodevelopment and pancreatic involvement. Sabrina Mechaussier et al. studied 7 CRSD patients from four unrelated families and found that patients had neurodevelopmental abnormalities and abnormal blood glucose levels when they had vision abnormalities caused by RIM2 mutation (Mechaussier et al., 2020). We mentioned above that RIM is expressed not only in the nervous system and audio-visual system, but also in the pancreas (Kelsell et al., 1998). *In vitro*, Rim2a participates in cAMP-enhanced insulin secretion through the cAMP-GEFII (a cAMP sensor, referred to as Epa2) pathway, suggesting that RIM2a plays a role in regulating insulin secretion (Ozaki et al., 2000). In type 2 diabetes, pancreatic β cells show glucose sensitivity defects, resulting in impaired insulin secretion. It was found that glucagon-like peptide-1 could enhance the activity of glucokinase in pancreatic cells through the combination of Epa2, Rim2, and Rab3A (Park et al., 2012). Furthermore, it was discovered that docking insulin vesicles required interaction between Rim2a and Rab3A, and the interaction between Rim2a and Munc13-1 activated Synaxin1, demonstrating that Rim2a provided favorable conditions for the fusing of insulin particles (Yasuda et al., 2010). Consistently, Photoreceptor synaptic transmission abnormalities, maternal behavior defects, and insulin resistance were all observed in RIM2^−/−^ mice (Schoch et al., 2006; Yasuda et al., 2010; Lohner et al., 2017).

### 6.3. Autism spectrum disorder

Asperger syndrome (AS) is a branch of autism spectrum disorder (ASD). It is a neurodevelopmental disorder characterized by difficulty interacting with others, impaired communication ability, and repetitive and stereotyped behavior patterns but no obvious intellectual impairment (Motlani et al., 2022). The involvement of *RIMS2* in autism spectrum disease (ASD) is supported by a genome-wide association study in affected individuals with Asperger syndrome, revealing a significant association with the *RIMS2*-associated SNP rs2080610 (Salyakina et al., 2010). RIM2 is listed as an ASD-related gene in the ASD database (Salyakina et al., 2010; Krishnan et al., 2016).

### 6.4. Degenerative lumbar scoliosis

DLS is a scoliosis deformity that occurs after bone maturation. Patients generally have no history of scoliosis or severe back pain. The etiology of DLS is not clear. A Korean population-based study assessed the potential role of RIM gene polymorphisms in bone degeneration. Two single-nucleotide polymorphisms (SNPs) (rs2028945 and rs10461) in the coding region of RIM2 in DLS were studied. The results suggested that RIM2 may affect the development of DLS. The rs10461 in RIMS2 was associated with DLS in the Korean population. However, a larger sample study is needed to confirm this finding (Kim et al., 2013).

## 7. Summary

RIM plays a role as a scaffold in the AZ and is closely linked to a variety of multidomain proteins, including RIM-BPs, Piccolo/Bassoon, ELKS/CAST, and Munc13-1. RIM affects the recruitment of presynaptic membrane VGCCs and affects the anchoring, priming, and fusion of SVs.

However, many questions need to be further explored. First, there is currently no complete picture of the dynamic interactions between CAZ molecules. Advances in imaging techniques may help to characterize the spatiotemporal dynamics of the interactions of RIM family members with molecules associated with vesicle release. Placing these molecules in a larger, more complex molecular environment could play an important role in the study of synaptic vesicle release mechanisms. Second, a previous article explained that RIM has multiple molecular functions, but each function is only partially lost when the gene is completely deleted. Moreover, after RIM was knocked out, vesicles were still released, which indicated that CAZ had a way to replace the RIM protein and that there might be redundancy between AZ components (Kaeser, 2011; Chien and Dickman, 2022). Therefore, the redundancy between RIM protein family subtypes and the functional redundancy between RIM proteins and presynaptic interacting molecules need to be further clarified. Third, the mechanism of RIM affecting synaptic plasticity remains to be further studied. Fourth, although some studies have proposed that the RIM gene is related to the pathogenesis of vision-related diseases, pancreatic diseases, and neuromuscular system diseases, the detailed pathogenic mechanisms need to be elucidated.

## Author contributions

SW, JF, XL, and DX: conceptualization. SW, JF, FT, LC, XZ, XL, and DX: software, validation, investigation, resources, and writing—review and editing. SW and JF: writing—original draft preparation. XL and DX: visualization and supervision. SW, JF, XL, and DX: funding acquisition. All authors have read and approved the final manuscript.

## Funding

This work was supported by the National Natural Science Foundation of China (82071353 and 82001593) and the key R&D projects of the Science and Technology Department of Sichuan Province (2021YFS0029 and 2020YFS0104).

## Conflict of interest

The authors declare that the research was conducted in the absence of any commercial or financial relationships that could be construed as a potential conflict of interest.

## Publisher’s note

All claims expressed in this article are solely those of the authors and do not necessarily represent those of their affiliated organizations, or those of the publisher, the editors and the reviewers. Any product that may be evaluated in this article, or claim that may be made by its manufacturer, is not guaranteed or endorsed by the publisher.

## Abbreviations

ASD: autism spectrum disorder
AZs: active zones
CAZ: cytomatrix in the active zone
CORD7: Cone-Rod Dystrophy 7
CRSDS: congenital nonprogressive cone-rod synaptic disorder syndrome
DLS: degenerative lumbar scoliosis
IHC: inner hair cells
NT: neurotransmitter
RIM: regulating synaptic membrane exocytosis protein
RIM-BPs: RIM-binding proteins
SNAP-25: synaptosomal-associated protein 25
SNARE: soluble N-ethylmaleimide-sensitive factor attachment protein receptor
SVs: synaptic vesicles
Syb2: synaptobrevin 2
Syx1: syntaxin-1
VAMP2: vesicle-associated membrane protein 2
VDCCs: voltage-dependent Ca2+ channels
